# A comparative study of semantic segmentation of omnidirectional images from a motorcycle perspective

**DOI:** 10.1038/s41598-022-08466-9

**Published:** 2022-03-23

**Authors:** Ahmed Rida Sekkat, Yohan Dupuis, Paul Honeine, Pascal Vasseur

**Affiliations:** 1 UNIROUEN, LITIS, Normandie Univ, Rouen, France; 2 LINEACT CESI, CESI, La Défense, Paris, France; 3 Laboratoire MIS, Université de Picardie Jules Verne, Amiens, France

**Keywords:** Computational science, Computer science

## Abstract

The semantic segmentation of omnidirectional urban driving images is a research topic that has increasingly attracted the attention of researchers, because the use of such images in driving scenes is highly relevant. However, the case of motorized two-wheelers has not been treated yet. Since the dynamics of these vehicles are very different from those of cars, we focus our study on images acquired using a motorcycle. This paper provides a thorough comparative study to show how different deep learning approaches handle omnidirectional images with different representations, including perspective, equirectangular, spherical, and fisheye, and presents the best solution to segment road scene omnidirectional images. We use in this study real perspective images, and synthetic perspective, fisheye and equirectangular images, simulated fisheye images, as well as a test set of real fisheye images. By analyzing both qualitative and quantitative results, the conclusions of this study are multiple, as it helps understand how the networks learn to deal with omnidirectional distortions. Our main findings are that models with planar convolutions give better results than the ones with spherical convolutions, and that models trained on omnidirectional representations transfer better to standard perspective images than vice versa.

## Introduction

With their large field-of-view, omnidirectional images are omnipresent in intelligent vehicles and robot navigation systems. At the same time, deep learning for computer vision has never been used as much as it is currently. However, computer vision algorithms used in these systems for tasks like scene understanding are mostly developed and tested for perspective conventional images captured using on-board cameras in cars. Furthermore, the case of motorized two-wheelers has not yet been studied while they present important differences in respect with cars. Indeed, in addition to distortions in omnidirectional images, these vehicles undergo rotations on the three axes, not like cars for example, which makes the semantic segmentation task even harder, due to the inadaptability of classical methods to changes of orientation without a particular treatment. Hence, the importance of optimizing these algorithms for omnidirectional imaging in general and for the case of motorized two-wheelers in particular. We can notice a recent growing interest in these algorithms dedicated to omnidirectional imaging. Several works treated the adaptation of existing algorithms or the development of new ones for tasks like object recognition and semantic segmentation on omnidirectional images, such as 360$^{\circ }$ and fisheye. In these two tasks, deep learning using convolutional neural networks (CNNs) on perspective images is the state-of-the-art solution. This is mainly thanks to the emergence of large-scale datasets of perspective images with ground truth annotation, such as CamVid^1^ and Cityscapes^2^. This convenience is not available for omnidirectional images and motorized two-wheelers. Until now, there is no available dataset of omnidirectional real urban driving images with ground truth for this kind of vehicle. To compensate for this major issue, several contributions on semantic segmentation of fisheye images for the case of cars work on data augmentation by training the state-of-the-art CNNs on perspective images that were deformed with a distortion simulating a fisheye effect^3–5^. On the other hand, some researchers proposed to encode directly the omnidirectional representation in the CNN^6^. More works proposed CNNs with deformable kernels^7,8^, or used icosahedron spherical image representation and spherical CNNs^9,10^.

More recently, researchers are considering the generation of synthetic images with realistic textures, thanks to simulators like CARLA simulator and Grand Theft Auto V (GTA V), which is a high-quality video game. The published OmniScape Dataset^11^ contains synthetic perspective, fisheye, catadioptric, and 360$^{\circ }$ urban driving images captured using a motorcycle with ground truth rendered from a virtual city and comes with pixel-level semantic annotation.

In this work, we take advantage of this dataset by building a comparative benchmark on it. We also used CamVid and Cityscapes, in addition to a test set of real fisheye images that we acquired and manually annotated, in order to study the performance of different semantic segmentation networks. This study consists in quantitative comparative analyses of the semantic segmentation task to take stock of research progress and answer the following questions:

- Is training networks developed for perspective representation on omnidirectional representations sufficient to have good results? Or, do we need to adapt CNNs for omnidirectional representations?
- Do networks learn a universal representation when trained on omnidirectional images? And what are their performances on perspective images in this case?
- Do networks with spherical convolutions give better results than the ones with planar convolutions?

In order to answer these questions, we conduct several experiments using a set of OmniScape synthetic images with perspective, fisheye, and equirectangular projections of the same scene taken from both front sides of a motorcycle, images from CamVid and Cityscapes dataset, and fisheye images from or real annotated test set. First, we test several semantic segmentation networks on CamVid images and choose the four networks that give the best results. We then make a cross-modality experiment. By modality we mean the pair Type/Representation, where the type of the image is either real or synthetic, and the geometry or the representation is either perspective, equirectangular, or fisheye. This cross-modality experiment is made by retraining the four networks separately on CamVid and Cityscapes images, OmniScape perspective, fisheye, and equirectangular images, to test them one by one with all these representations, as well as on our test set of real fisheye images. We use two models based on icosahedral representation dedicated to spherical images to perform semantic segmentation using the same equirectangular images used in the previous experiments. In the end, this allows us to conclude on the efficiency of different neural networks dedicated to semantic segmentation of perspective images on equirectangular and fisheye images, as well as the performance of these networks when trained on omnidirectional images. Finally, the relevance of the two icosahedral-based models is compared to the best planar model for equirectangular images. Studies made on semantic segmentation of real fisheye images rarely present quantitative results, due to the scarcity of dataset that contains omnidirectional urban driving images ground truth. In this study, we present quantitative results in addition to qualitative ones.

The remainder of this paper is organized as follows. “Related work” section presents different works on semantic segmentation of omnidirectional images. “The experimental approach” section introduces our experimental approach. “Results and discussions” section presents the results obtained and discusses them. Finally, “Conclusion and future work” section concludes the paper.

## Related work

Distinct studies were carried out on semantic segmentation of omnidirectional images to compensate for the lack of algorithms dedicated to this type of data, as succinctly presented in this section.

### Fisheye images

Fisheye cameras have a field of view that can reach 180°. Since CNNs for semantic segmentation are not designed for these images, and due to the scarcity of fisheye datasets with ground truth, researchers worked on the deformation of conventional images from Cityscapes or SYNTHIA^12^, by applying distortion to simulate the fisheye effect^3–5,13^. The used distortion is described by $r_{p}=f\tan (r_{f}/f)$, which represents the mapping from the fisheye image point $P_{f}=\left( x_{f}, y_{f}\right)$ to the perspective image point $P_{p}=\left( x_{p}, y_{p}\right)$, where $r_{p}^2={\left( x_{p}-u_{p x}\right) ^{2}+\left( y_{p}-u_{p y}\right) ^{2}}$ is the square distance between the image point $P_{p}$ and the principal point $U_{p}=\left( u_{p x}, u_{p y}\right)$ in the perspective image, and $r_{f}^2={\left( x_{f}-u_{f x}\right) ^{2}+\left( y_{f}-u_{f y}\right) ^{2}}$ denotes the square distance between the image point $P_{f}$ and the principal point $U_{f}=\left( u_{f x}, u_{f y}\right)$ in the fisheye image. This distortion only depends on the focal length *f*; thus, several focal lengths were set to simulate different fisheye images with their corresponding annotations. Using the images resulting from this transformation, Deng et al.^4^ proposed OPP-net based on an Overlapping Pyramid Pooling module. Saez et al.^13^ proposed an adaptation of Efficient Residual Factorized Network (ERFNet)^14^ to fisheye road images in order to achieve real-time semantic segmentation and tested it on real fisheye images, but only qualitative results were exposed. Deng et al.^5^ used the same method to achieve road scene semantic segmentation of fisheye surround-view cameras using restricted deformable convolution. The networks were trained on data from Cityscapes and SYNTHIA datasets and tested on real fisheye images.

### Panoramic images

Xu et al.^15^ used synthetic images captured from SYNTHIA to create a dataset of panoramic images by stitching images taken from different directions. Using these images, the authors show that panoramic images improve segmentation results. Yang et al.^16^ proposed a panoramic annular semantic segmentation framework (PASS), such as the cited works for fisheye images, they made a data augmentation method by adding distortion to perspective images in the training set. They then used planar CNNs after unfolding and partitioning the panoramic images. Ma et al.^17^ addressed the problem of semantic segmentation of panoramic images via an unsupervised domain adaptation method from perspective to panoramic images. Orhan et al.^18^ achieved the same task as Ma et al.^17^ by proposing a network that uses deformable convolution where the offsets added to the kernel location are not learned but computed using the geometry of the equirectangular projection. Orhan et al.^18^ have also shared an outdoor annotated panoramic image dataset.

### Equirectangular images

Equirectangular representation is the most popular projection for 360$^{\circ }$ images thanks to the simple transformation from spherical coordinates into planar coordinates. Classical CNNs designed for perspective images can be used for data under the equirectangular representation. But spherical input suffers from distortion in polar regions. Different approaches were proposed to handle this issue. Monroy et al.^19^ proposed SalNet360 where omnidirectional images are mapped to cubemap projection and trained using planar CNNs to predict visual attention. However, artefacts are created when recombining the cubemap faces to omnidirectional image. Lai et al.^20^ used semantic segmentation of equirectangular images to convert panoramic videos to normal perspective images. However for this task, highly accurate semantic segmentation was not required, a frame-based fully convolutional network FCN was used in^21^. Su et al.^22^ translated a planar CNN to process 360$^{\circ }$ images directly in the equirectangular projection for object detection. And in^23^ they proposed the kernel transformer network (KTN) to transfer convolution kernels from perspective images to equirectangular projection of 360$^{\circ }$ images. Tateno et al.^24^, proposed a learning approach for equirectangular images using a distortion-aware deformable convolution filter for depth estimation from a single image; this approach was also demonstrated on 360$^{\circ }$ semantic segmentation.

### Spherical representations

Because of distortions resulting from the equirectangular representation, most recent studies on this topic choose to work on the spherical representation. Cohen et al.^25^ developed spherical convolutions by replacing the translations in the plane with rotations of the sphere. Other studies took advantage of a more accurate discretization of the sphere, namely the icosahedral spherical approximation. The discretization of the sphere is represented by a spherical mesh generated by subdividing each face of a regular icosahedron into four equal triangles. Lee et al.^26^ proposed an orientation-dependent kernel method regarding triangle faces. This method was demonstrated through classification, detection, and semantic segmentation. Zhang et al.^27^ also addressed semantic segmentation on omnidirectional images using icosahedron spheres by proposing an orientation aware CNN framework. Jiang et al.^10^ proposed UGSCNN to train spherical data mapped to an icosahedron mesh, by replacing conventional convolution kernels with linear combinations of learnable weighted operators. Kumatsu et al.^28^ addressed a method for all-around depth estimation from multiple omnidirectional images by proposing a new icosahedron-based convolution named CrownConv. Cohen et al.^29^ proposed gauge equivariant convolutional networks on manifolds and demonstrated its relevance by achieving semantic segmentation. Eder et al.^9^ proposed Tangent-images, which is a spherical image representation that consists in rendering these images to a set of locally planar images grids tangent to a subdivided icosahedron; planar convolutions can be then used on the resulting images to achieve different computer vision tasks.

We can notice that in general there are two groups of works, the first one uses planar convolutions and the second one uses convolution on manifolds. In the next section, we detail the experimental approach we followed in our work to make a fair comparison between the main semantic segmentation solutions proposed in the state-of-the-art.

## The experimental approach

To answer the questions addressed in the introduction, we carried out different experiments. We choose to use four networks developed for perspective images as well as UGSCNN and Tangent-images, which use the icosahedral manifold. One of the reasons why we choose to use UGSCNN and Tangent-images in addition to being the state-of-the-art solutions that use the icosahedral manifold is the availability of the source code. In the first experiment, we did a selection to choose the networks we will use in this study, and to choose the size of the data-set we made a performance versus number of samples experiment. Then we made a cross-modality experiment by training the four selected networks on real CamVid and Cityscapes perspective images and fisheye, equirectangular, and perspective OmniScape synthetic images. We also trained the networks on transformed Cityscapes images with the same transformation explained in section related work on fisheye images. In addition, we mixed transformed Cityscapes images with OmniScape images in the training set. We tested the trained networks on all these modalities and also on our test set of 15 fisheye images. To evaluate the quality of results, we performed a leave-one-out cross-validation experiment on this set. In the last experiment, we trained UGSCNN^10^ and the baseline used in, as well as Tangent-images representation with the same networks proposed in^9^ on the same OmniScape equirectangular images used in the second experiment, we tested it on the same modality with different resolutions to compare the results with the best model for equirectangular images in the second experiment. In all the experiments, we used RGB images with 15 semantic classes. It is worth noting that all the networks in this study are trained for 300 epochs and from scratch without data augmentation and domain adaptation modules. We directly take the hyper-parameters proposed in their respective publications. We do not fine-tune each network since this is not the purpose of the paper. In all the following experiments, we will use two metrics, the mean accuracy (mAcc) and the mean intersection over union (mIOU). The accuracy and the intersection over union are computed for each class separately, and then averaged over all classes to provide a global mean accuracy and mean intersection over union scores of the semantic segmentation predictions. A single GPU NVIDIA Tesla V100 SXM2 was used in all the experiments.

**Table 1:** Results of the networks selection using real perspective images from CamVid dataset (%).

	mAcc	mIoU	Per-class Acc														
			Void	Sky	Building	Fence	Other	Person	Pole	Road line	Road	Sidewalk	Vegetation	Two wheeled	Four wheeled	Wall	Traffic sign
FC-DenseNet56^30^	91.8	60.3	46.4	96.7	90.5	75.8	63.3	58.5	41.3	97.0	97.9	**90.5**	88.3	73.6	89.1	76.4	55.1
FC-DenseNet67^30^	92.3	54.4	47.7	**96.8**	92.2	**78.9**	**67.5**	62.7	**54.4**	96.9	**98.3**	88.6	87.7	73.9	**89.9**	77.1	**60.8**
**FC-DenseNet103**^30^	**92.2**	**62.0**	49.4	96.7	91.7	78.5	65.4	57.2	46.3	97.4	98.2	90.2	**88.4**	72.7	89.7	77.3	55.0
MobileUNet$^{\star }$^31^	87.6	48.9	37.0	93.6	87.1	73.4	53.2	33.6	15.0	96.5	96.8	83.2	83.0	62.6	80.1	66.4	34.6
PSPNet^32^	89.0	54.6	38.9	95.7	89.8	74.6	60.6	55.9	34.5	95.5	97.6	84.5	83.5	67.2	86.5	71.9	50.9
GCN^33^	90.7	56.2	42.1	96.3	90.5	71.5	52.2	53.6	40.5	96.0	97.9	89.7	86.0	66.0	83.6	74.1	49.4
**FRRN**^34^	**91.9**	**61.8**	46.4	96.6	92.2	78.0	66.3	**64.9**	49.4	97.5	**98.3**	89.9	86.7	72.7	89.4	**77.6**	57.9
DeepLabV3^35^	86.8	47.1	33.3	94.1	89.9	70.9	51.7	32.6	17.0	94.0	96.9	80.8	80.8	62.1	76.2	62.4	33.9
DeepLabV3+^36^	89.3	53.2	39.7	95.1	89.5	72.6	53.8	45.4	33.0	94.4	97.8	86.6	87.1	64.2	84.0	68.5	45.5
**RefineNet**^37^	**91.2**	**59.3**	42.9	96.0	**92.5**	75.5	60.6	57.0	39.8	**97.7**	98.1	89.1	87.4	71.0	86.3	74.5	51.9
AdapNet$^{\star }$^38^	87.3	47.9	38.6	96.7	89.2	71.9	52.8	26.5	18.3	96.3	96.2	78.3	80.1	61.0	76.8	65.6	34.5
DenseASPP^39^	87.9	50.6	39.5	91.4	90.5	71.4	54.9	41.3	23.9	94.8	97.6	83.1	82.2	65.2	78.1	67.7	37.4
BiSeNet$^{\star }$^40^	90.3	55.1	40.2	95.9	90.6	74.6	53.7	47.0	24.9	96.9	97.9	88.2	87.6	65.8	85.3	70.6	50.6
**SegNet**$^{\star }$^41^	**92.0**	**61.8**	**50.1**	96.2	92.1	78.5	66.5	59.3	46.3	97.5	98.0	89.5	88.0	**74.3**	89.0	76.6	57.0

$^{\star }$Designed or can be used in real-time.

The bold font shows the scores (mIoU and accuracies) of the four chosen networks, and the best accuracies obtained per class.

### Networks selection

**Figure 1:**
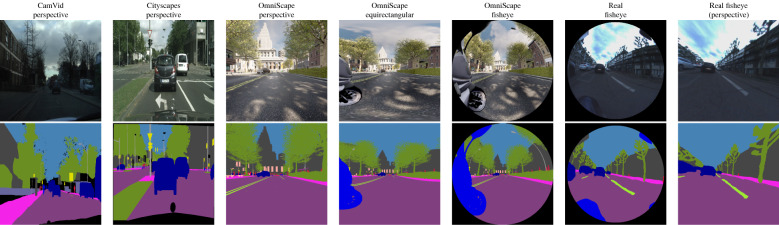
Modalities used and corresponding semantic segmentation ground truth.

The goal of this experiment is to choose the four most relevant networks that we will use in the cross-modality. To choose these networks, we made a selection using real perspective images from CamVid Dataset among 11 networks representing different architectures proposed in the state-of-the-art on semantic segmentation of perspective images. The obtained results are listed in Table 1. We trained and tested all the networks on the same sets of $512\times 512$ CamVid images. We used 700 images, 420 in the training set, 112 in the validation set, and the remaining 168 images in the test set. The images are segmented into 32 object classes. We mapped similar classes into 15 to have the same classes present in OmniScape. Figure 1 shows a CamVid image with ground truth.

Some networks rely on a pre-trained ResNet for feature extraction. Pre-trained ResNet weights are then downloaded and used in this case. These networks are: PSPNet^32^, RefineNet^37^, DeepLabV3^35^, DeepLabV3+^36^, GCN^33^.

The results of this first selection are presented in Table 1. We can notice that all networks are quite similar in general. Indeed, some small changes in training parameters may change the ranking, but we will likely obtain close results anyway. The contribution is not to optimize and try to get the best results for each of the networks used in the study. The idea is to use them as they are presented and published. However, the four networks that give the best mIoU score with good mAcc are Fully Convolutional DenseNet, Full-Resolution Residual Network, SegNet, and RefineNet. For the Fully Convolutional DenseNet network, we chose to use just the architecture built from 103 convolutional layers for the next experiment. In the following, we present a brief overview of each of the four chosen networks.

- The Fully Convolutional DenseNet^30^ is an adaptation of DenseNets for semantic segmentation. It is a U-Net architecture where the convolutional layers are replaced with dense blocks. Each convolution layer is then directly connected to every other layer. This network has 9M parameters.
- The Full-Resolution Residual Network^34^ combines two distinct processing streams. One stream undergoes a sequence of pooling operations and is responsible for understanding large-scale relationships of the elements in the image. The second stream carries feature maps at the full image resolution, giving a precise adherence to boundaries. The pooling operations in the first stream act like residual units for the second and carry high level information over the network.This network has 17M parameters.
- The SegNet^41^ consists of an encoder–decoder layer followed by a pixel-wise classification layer. The architecture of the encoder layer is identical to the VGG16 network. Each encoder consists of one or more convolutional layers. This layer contains batch normalization, a ReLU non-linearity, a non-overlapping max-pooling, and sub-sampling. This network has 35M parameters.
- The RefineNet^37^ is considered as a generic multi-path refinement network that uses long range residual connections to enable high resolution prediction by exploiting all the information available in the down-sampling process. By using fine-grained features from earlier convolution, the deeper layers that capture high level semantic features can be directly refined. This network has 85M parameters.

**Table 2:** Image sets and networks used in the cross-modality experiment.

Training sets	Testing sets	Networks
CamVid	CamVid	FC-DenseNet103
OmniScape Perspective images	OmniScape Perspective images	SegNet
OmniScape Fisheye images	OmniScape Fisheye images	FRRN
OmniScape Equirectangular images	OmniScape Equirectangular images	RefineNet
Cityscapes	Cityscapes	
	Real Fisheye images	
	Real Fisheye images (perspective)	

### Performance versus number of samples

**Figure 2:**
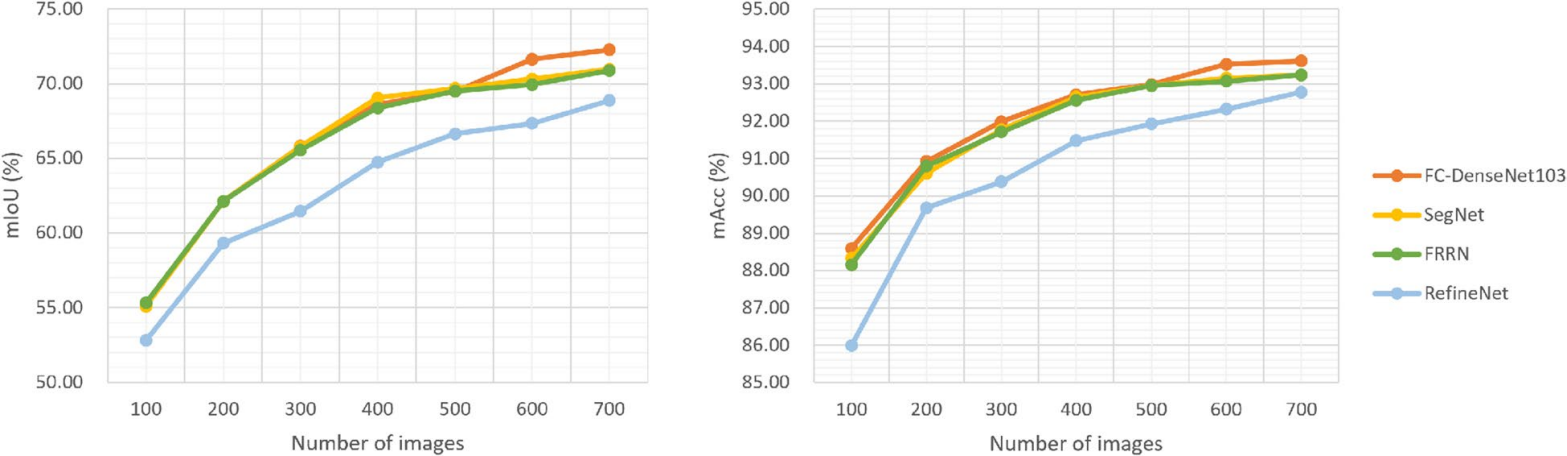
mIoU and mAcc versus number of samples used in the training set.

We used the four selected networks and CamVid dataset with 100 to 700 images, using each time 60% in the training set, 24% in the validation set and 16% images in the test set. Figure 2 shows the evolution of the mAcc and mIoU when the size of the training set increases. We can see that the performance does not improve beyond 500 images. We have decided then to keep using 700 images in all the experiments.

### Cross-modality experiment

In this experiment, we used 700 captures from OmniScape, 700 images from CamVid, Cityscapes, and 15 images from our real fisheye images test set. The OmniScape Dataset provides synthetic omnidirectional images, namely 360$^{\circ }$ equirectangular, fisheye, and catadioptric stereo RGB images from the two front sides of a motorcycle with semantic segmentation and depth map ground truth. The images in OmniScape are annotated into 15 classes. For Equirectangular representation, we crop the images to keep just 180$^{\circ }$, which represents the front side, so all modalities can be fairly compared to each other. Figure 1 shows OmniScape different modalities used with semantic segmentation ground truth. Our test set contains real fisheye images, we also use these images under the perspective representation with a FOV 126$^{\circ }$. These real fisheye images are captured using the same disposition used in the OmniScape dataset; Stereo fisheye cameras placed in the two front sides of a motorcycle. We annotated 15 different images into 15 classes like the OmniScape dataset, using the open source tool for annotation PixelAnnotationTool^42^. Figure 1 shows an example of images from this set with ground truth. We split the 700 images of each modality like a standard cross validation problem into three sets: a training set of 420 images, a validation set of 112 images, and a test set of 168 images. We trained the four chosen networks on OmniScape images using fisheye, perspective, and 180$^{\circ }$ equirectangular images and also CamVid and Cityscapes. Then, we tested all the trained networks on all these modalities, and on our test set of fisheye real images annotated manually. The class Void in CamVid represents far objects that are undefined, and in the OmniScape dataset, it represents the dark space surrounding the fisheye image. In this experiment, we dropped this class and we did not take it into account in the evaluation of the scores because it does not represent a piece of information. In Table 2 are listed the training and test sets along with the networks used in the cross-modality.

### Leave-one-out experiment

In this experiment we trained the four networks on the 15 real fisheye images by leaving one image out each time to test the networks on it, resulting in 15 training sets. The purpose of the experiment is to have an idea about the performance while using other modalities in the training set.

### Distorted perspective images experiment

**Figure 3:**
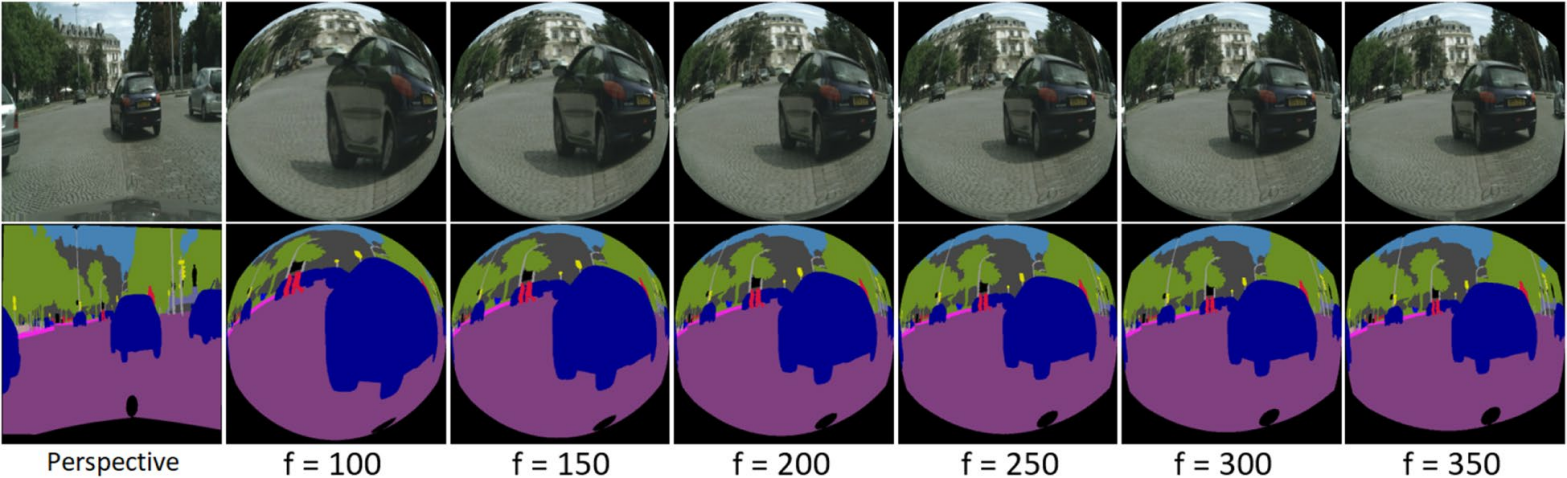
Examples of the distorted perspective Cityscapes images used.

In this experiment, we trained the networks on transformed Cityscapes images with the same transformation explained in section related work on fisheye images and used by several previous researchers^3–5,13^. We choose to use six focal lengths *f* (100, 150, 200, 250, 300, 350) to cover all the values used in previous studies. The images of these sets are shown in Figure 3. We then used training sets where the types of representation are mixed and contain real and synthetic images, to explore if the networks can learn on different modalities and improve the results. We used Cityscapes images with and without tangent transform and OmniScape images. We created seven sets, each having 50% of OmniScapes and 50% of Cityscapes images, with and without tangent transformation; we denote these sets OmniCityscapes. In this experiment, only the real fisheye images are used as the test set. It is worth noting that the unpleasant effect of using a tangent transformation is that the field of view of these images is not comparable to the large field of view of omnidirectional images. The amount of information is much more important in real omnidirectional images than in images generated with this transformation. We can also easily notice in Figures 1 and 3 that pixels representing foreground object classes, like Person and Vehicle for example, are very less in real fisheye images compared to transformed perspective images.

### Comparison with icosahedral-based CNNs

**Figure 4:**
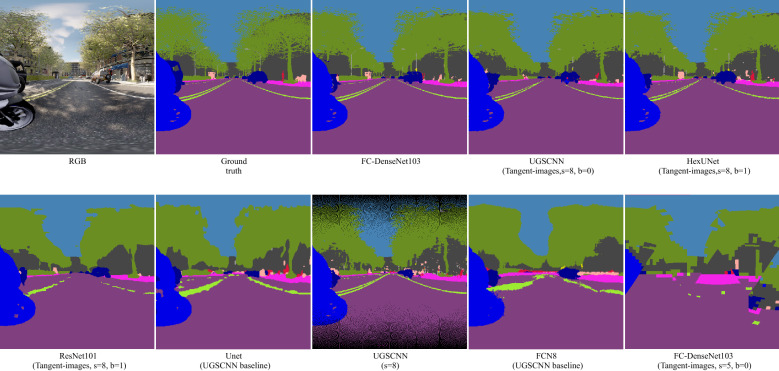
Predicted equirectangular images using icosahedral-based networks.

The motivation behind this experiment is to know if icosahedral-based convolution gives better results than planar networks, especially the ones used in the second experiment when tested on equirectangular images. The idea behind this comparison is to highlight the imperfections for possible improvement and to know what is better to segment equirectangular images. In this experiment, we used UGSCNN and Tangent-images representation. We trained UGSCNN on the same OmniScape equirectangular images used in the cross-modality experiment. Since the resolution of the images used is $512\times 1024$, we performed this experiment using level 8. We used in this experiment just RGB, without depth map since the depth map was not used by the other networks. The network is trained with a batch size 8 for level 8. We used like in^10^ the weighted cross-entropy loss for training and zero weight for the dropped class Void. To display qualitative results, we unwrap the sphere using the UV mapping process. The equirectangular images are regenerated using the following for any point *P* on the sphere:

1 $$\begin{aligned} u=0.5+\frac{{{{\mathrm{arctan2}}}}\left( d_{z}, d_{x}\right) }{2 \pi }, \qquad v=0.5-\frac{\arcsin \left( d_{y}\right) }{\pi }, \end{aligned}$$

where (*u*, *v*) are the coordinates in the equirectangular image in the range [0, 1], and ${d=(d_{x}, d_{y}, d_{z})}$ the unit vector from *P* to the sphere’s origin. Figure 4 [UGSCNN(s = 8)] shows one example of unwrapped equirectangular image from a sphere. We also used for comparison the same baseline networks used in UGSCNN article, namely UNet^43^ and FCN8s^21^ with the same equirectangular images.

UGSCNN is an orientation-aware method. In this network, the convolution kernel is replaced by linear combinations of differential operators that are weighted by learnable parameters using standard back-propagation. The operators are estimated on unstructured grids.

Tangent-images is a representation where spherical data are projected into square oriented pixel grids tangent to the sphere according to the faces of an icosahedron. We used this representation with three levels (s) 5, 7, and 8, and three base subdivisions (b), 0, 1, and 2, to train the same networks proposed in the Tangent-images article, namely HexUNet^27^, UGSCNN where the specific convolution kernel was replaced by a $3\times 3$ 2D convolution and ResNet101^44^, as well as the best model achieving best results trained and tested on equirectangular images in the first experiment FC-DenseNet103. In the next section, we will present the combination of level and base subdivisions that gives the best results for each used network.

## Results and discussions

**Figure 5:**
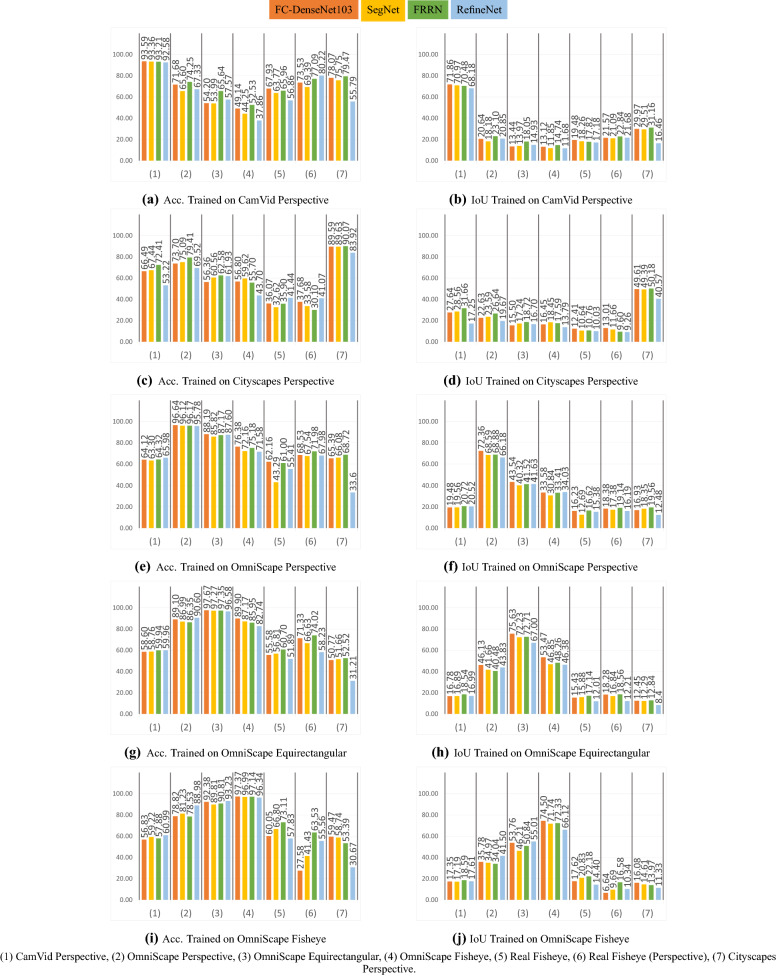
Per test set mAcc and mIoU obtained in the cross-modality experiment (%).

In this section, we present the results of all the experiments explained above and the comparison with icosahedral-based CNNs. We discuss and give quantitative results, as well as qualitative ones. We answer the questions raised in the introduction by analyzing the obtained results. And finally, we make a comparison between the combinations network/training-set, which gives the best results on equirectangular images in the first experiment and icosahedral-based solutions UGSCNN and Tangent-images. Figure 5 represents an overview of the results obtained in the cross-modality experiment using clustered columns. It summarizes all the results obtained by 140 testing processes. As a first remark, we can see that the best results are always obtained when the dataset does not change between training and testing processes. The four networks are very sensitive to texture changes. We see that when the environment changes (real versus synthetic), the performance deteriorates drastically. This problem could be viewed as a domain adaptation one but it is not the aim of this study.

### Omnidirectional images

The four networks, when trained on fisheye images or equirectangular images and tested on the same modalities, give a mAcc not less than 90% and a mIoU higher than 66% without exception. It shows that networks designed for perspective images give good results when trained and tested on omnidirectional ones. This answers the first question in the introduction: The network architectures that were proposed for perspective images can be used for omnidirectional images after necessary retraining phases, and possibly using some adjustments like the input size and the aspect ratio of the images. These architectures can then achieve similar performance on both representations, perspective and omnidirectional.

**Table 3:** Results obtained in the leave-one-out experiment (%).

	mAcc	mIoU	Network
Max	97.95	65.30	FRRN
Min	47.50	18.37	RefineNet
Mean	87.17	39.06	

### Real fisheye images

**Figure 6:**
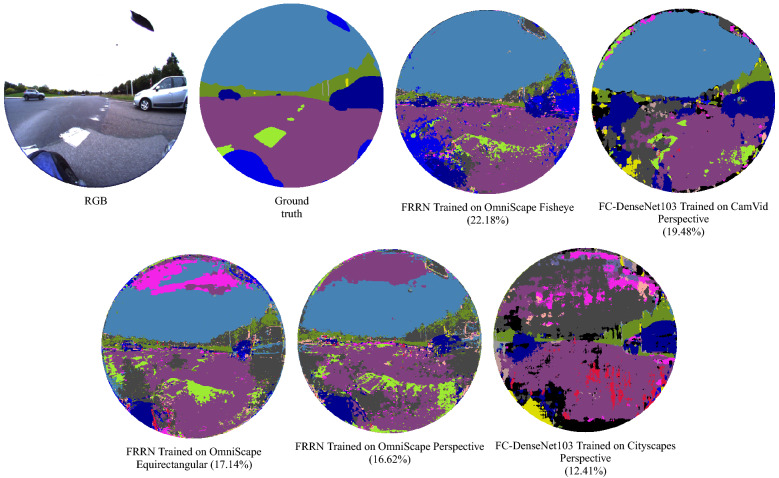
Qualitative results on a real fisheye image using networks given best mIoU for each modality in the cross-modality experiment.

**Figure 7:**
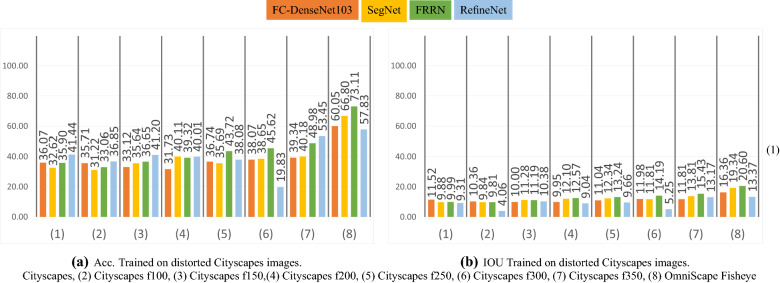
Per test set mAcc and mIoU obtained using distorted Cityscapes images (%).

**Figure 8:**
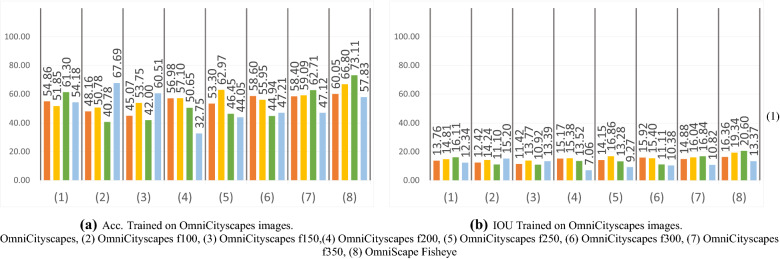
Per test set mAcc and mIoU obtained using OmniCityscapes images (%).

On one hand, the results obtained for real fisheye images are poor, the highest obtained mIoU being 22.18% with mAcc equal to 73.11%. On the other hand, as listed in Table 3 the mIoU obtained in the leave-one-out experiment is 39.06% and the mAcc is 87.17%, representing unbiased results with the least variability. Considering the mean of the leave-one-out result as the best results we could expect using these images in the test set, we can consider that results obtained for real fisheye images in the cross-modality experiment are finally encouraging. The best results are reached when OmniScape fisheye images were used in training, using FRRN the same network with best results in the leave-one-out experiment. On the flip side, we can notice that in general there is not a big gap between the results obtained when testing on real fisheye images and when testing on the same images under the perspective representation, the best mIoU for real fisheye images under the perspective representation being 21.21% when trained on CamVid. When the networks are trained on fisheye OmniScape images, we obtain inferior results on the perspective representation but the best results on the fisheye representation. This confirms that it is thanks to the geometry of fisheye OmniScape images that the results are better since the intrinsic parameters of these images are the same as the real fisheye camera. Figures 6 and 7 shows results obtained in the distorted perspective images experiment and results obtained when we mix real Cityscapes images with synthetic OmniScape fisheye images. On one hand, we observe that the tangent transformation did not give better results than OmniScape fisheye images; however, the results are better than Cityscapes without transformation especially with f = 350, which represents the images with the least deformation. On the other hand, the mixed training sets did not achieve better than not mixed sets, but the mixing improved the results of transformed Cityscapes images especially for RefineNet f=100 and SegNet f = 250; it shows that both textures and geometry are important, but the geometry slightly outweighs the texture in this case. The use of Real fisheye or more photorealistic fisheye images in the training could improve the results. Figure 8 shows qualitative results obtained by the best networks for each modality. It is worth noting that the accuracy and the intersection over union are computed without taking into account the surrounding black area in fisheye images. We consider just the part that contains the information. FRRN with OmniScape fisheye images gives the best results when testing on real fisheye images. However, it is not the fastest in terms of computation time as shown in Table 4.

**Table 4:** Runtime of the selected networks for OmniScape equirectangular images using NVIDIA Tesla V100 SXM2.

Network	Training runtime (h)	Testing average runtime (ms)
SegNet	10.93	263.4
RefineNet	14.97	271.6
FRRN	15.11	349.6
FC-DenseNet103	15.8	795.2

### OmniScape images

**Figure 9:**
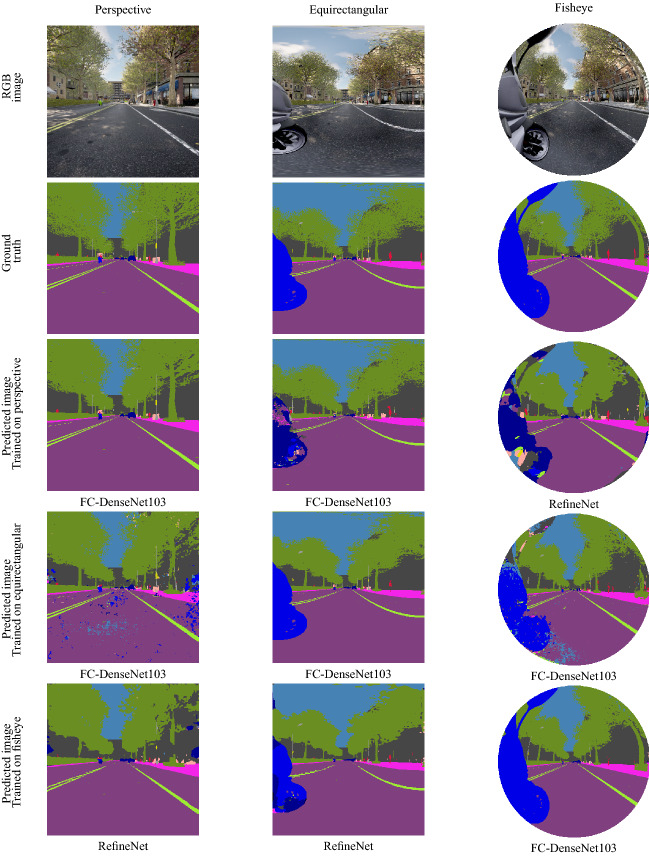
Qualitative results for networks with best mIoU in the cross-modality experiment for OmniScape images.

In these images, the only difference is the camera itself since the same scene is captured by three cameras: perspective, fisheye, and 360$^{\circ }$ equirectangular. This configuration allows us to make a fair comparison between all these modalities. We notice that the best results are obtained when the training and testing sets are the same as shown in gray in Tables 5 and 6 that list for each combination the best network in terms of mAcc and mIoU. When the training and testing sets are not the same, we can notice that omnidirectional images (fisheye and equirectangular) are more robust and can learn a universal representation better than when trained on perspective images. We can also notice that FC-DenseNet103 and RefineNet achieve the best results, but sometimes they are just slightly better than the others. Figure 9 shows an example of qualitative results for all the combinations.

**Table 5:**
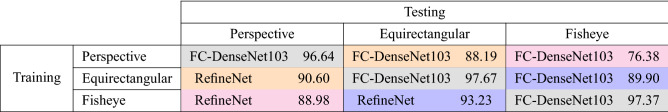
Networks with best mAcc (%) in the cross-modality experiment for OmniScape images.

**Table 6:**
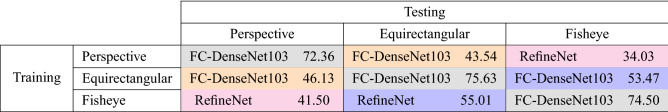
Networks with best mIoU (%) in the cross-modality experiment for OmniScape images.

### Equirectangular images

**Figure 10:**
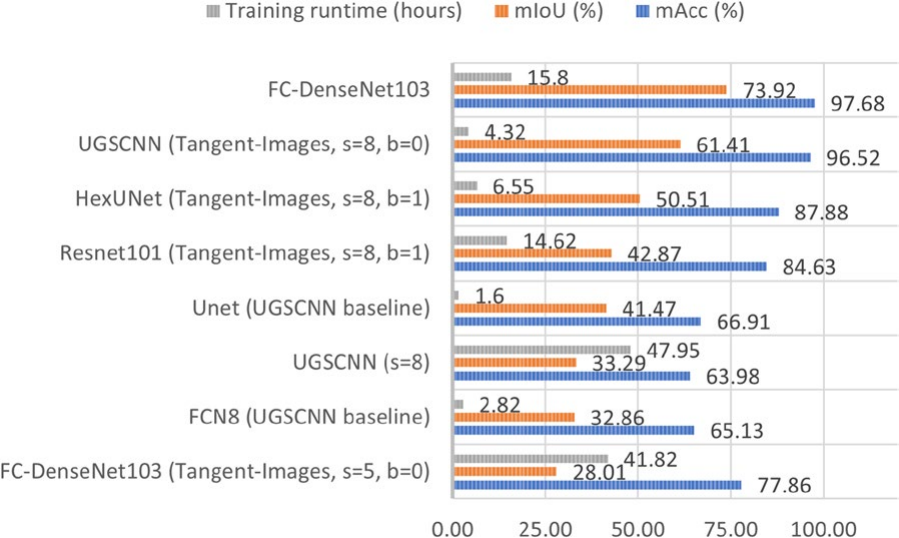
mAcc, mIoU and training runtime using icosahedral-based networks.

We saw in the previous experiment that FC-DenseNet103 gives the best results when trained and tested on equirectangular. This becomes our baseline in this section, where we seek to discover if icosahedral-based networks can achieve better results or not. Figure 10 presents the best combination of levels (5, 7, 8) and base subdivision (0, 1, 2) for each network used with Tangent-images, and UGSCNN level 8, the baseline used by UGSCNN authors, as well as FC-DenseNet103. Figure 4 shows the corresponding qualitative results. The combinations of Tangent-images with FC-DenseNet103 using s = 7 and s = 8 were stopped because it needed several days of training. This is due to the fact that FC-DenseNet103 has several layers, and the Tangent-images representation results in a big number of images.

We notice that Unet outperforms UGSCNN but both, as well as FCN8, are far behind FC-DenseNet103 which is the best. We observe that Tangent-images improve the performance of UGSCNN; this can be explained by the fact that the convolution in this case was replaced by a 2D convolution. We also observe that the best results for Tangent-images are obtained when using level 8 with b = 0 or b = 1, which means Tangent-images with $256 \times 256$ and $128 \times 128$ pixels. We can deduce that Tangent-images is not useful in our case, even if it can be for high-level resolution images^9^. And also using a planar 2D convolution is better, since it enhances the results obtained by UGSCNN. Finally, we can deduce that using a network based on planar convolution is better than networks with icosahedral based convolution for our use case.

### Summary

To summarize all the experiments conducted in this work, we can say that semantic segmentation networks made for perspective images give good results and are more robust when trained on omnidirectional images. They are able to learn a universal representation and achieve better results on all modalities than if trained on perspective images. Finally, we made a comparison between a network that uses icosahedral-based networks and a network with planar convolutions using equirectangular images. Working with the icosahedral manifold is very greedy in terms of computation time and memory, but does not necessarily give better results. We saw that a network based on planar convolution trained on equirectangular images is sufficient and outperforms icosahedral-based networks in segmenting road scene equirectangular images.

## Conclusion and future work

This paper takes stock of progress made on semantic segmentation of omnidirectional images. We presented a comparative study of semantic segmentation using equirectangular, fisheye, and perspective images, from real and synthetic datasets. By comparing different networks of semantic segmentation, we proved that networks developed for perspective images with planar convolutions when trained on omnidirectional images give good results and they are more robust against modality changes. We also made a comparison using equirectangular images with both planar convolution and different icosahedral-based solutions. The experiments show that planar convolution is better. As we noticed that networks used are sensitive to textures and environment changes, one solution can be to use networks performing image to image translation like pix2pix^45^ to generate more realistic images using the OmniScape dataset since we lack datasets of real omnidirectional images with ground truth especially for the case of motorized two-wheelers. Ideally, a network using an equivariant convolution able to learn shapes and geometry of objects regardless of texture and position on the omnidirectional image would be more adequate for omnidirectional images. This can be achieved by using convolution on manifold as shown by Cohen et al.^29^. The works done on the icosahedral representation are encouraging, but for now, the experiments showed that planar convolution is better for the task of semantic segmentation of omnidirectional road scenes images.
